# Supplementary material for: Content-rich biological network constructed by mining PubMed abstracts
Source: BMC Bioinformatics. 2004 Oct 8;5:147. doi: 10.1186/1471-2105-5-147 (PMC528731; doi:10.1186/1471-2105-5-147)
Supplement: Additional File 2 — The original results of the above study (non-essential files are deleted to keep the file size under the limit set by BMC bioinformatics). [file 1471-2105-5-147-S2.bz2 › chilibotAdditionalFile2/dip10/368ID9045692E6042/html/FGFR3_FGF4.html]

 


 **FGFR3** and **FGF4** 
  
Found 17 abstracts in PubMed, retrieved 10.  
 

 What does Google say? 
 PDF only 
| .edu only 

---

**Interactive relationship** (e.g. stimulation, inhibition, etc)

**Non-interactive relationship** (e.g. studied together, co-existance, homology, etc.)

- In addition, the growth status of the KMS 11 and KMS 18 lines with FGF 1 or anti FGF 4  [ **FGF4** ]  neutralizing monoclonal antibody MoAb supplementation was investigated because FGF 1 and 4 are known as the principal ligands for  **FGFR3** .  Ref: 10568829 Int J Oncol, 1999
- 3 ; q32.3 may provide myeloma cells with a growth advantage via an autocrine mechanism between  **FGFR3**  and FGF 4  [ **FGF4** ] .  Ref: 10568829 Int J Oncol, 1999
